# *Rhodococcus opacus* B4: a promising bacterium for production of biofuels and biobased chemicals

**DOI:** 10.1186/s13568-016-0207-y

**Published:** 2016-05-14

**Authors:** Ana Rita Castro, Isabel Rocha, Maria Madalena Alves, Maria Alcina Pereira

**Affiliations:** CEB–Centro de Engenharia Biológica, Universidade do Minho, Campus de Gualtar, 4710-57 Braga, Portugal

**Keywords:** *Rhodococcus opacus*, Lipids, Hexadecane, Triacylglycerols, Fatty acids, Biodiesel

## Abstract

Bacterial lipids have relevant applications in the production of renewable fuels and biobased oleochemicals. The genus *Rhodococcus* is one of the most relevant lipid producers due to its capability to accumulate those compounds, mainly triacylglycerols (TAG), when cultivated on different defined substrates, namely sugars, organic acids and hydrocarbons but also on complex carbon sources present in industrial wastes. In this work, the production of storage lipids by *Rhodococcus opacus* B4 using glucose, acetate and hexadecane is reported for the first time and its productivity compared with *Rhodococcus opacus* PD630, the best TAG producer bacterium reported. Both strains accumulated mainly TAG from all carbon sources, being influenced by the carbon source itself and by the duration of the accumulation period. *R. opacus* B4 produced 0.09 and 0.14 g L^−1^ at 24 and 72 h, with hexadecane as carbon source, which was 2 and 3.3 fold higher than the volumetric production obtained by *R. opacus* PD630. Both strains presented similar fatty acids (FA) profiles in intact cells while in TAG produced fraction, *R. opacus* B4 revealed a higher variability in fatty acid composition than *R. opacus* PD630, when both strains were cultivated on hexadecane. The obtained results open new perspectives for the use of *R. opacus* B4 to produce TAG, in particular using oily (alkane-contaminated) waste and wastewater as cheap raw-materials. Combining TAG production with hydrocarbons degradation is a promising strategy to achieve environmental remediation while producing added value compounds.

## Introduction

In the last years, attention has been paid to microbes as lipid producers for biotechnological and industrial applications. Bacterial lipids such as triacylglycerol (TAG–triesters of glycerol and long-chain fatty acids) and wax esters (WE–esters of primary long-chain fatty acids and primary long chain fatty alcohols), have relevant applications in the production of food additives, cosmetics, lubricants, oleochemicals, candles and biofuels (Röttig et al. 2010; Holder et al. 2011). The majority of the bacterial species synthesizes polyhydroxyalkanoates (PHA) as storage compounds (Alvarez et al. 1997a; Steinbüchel and Hein 2001), whereas the ability to accumulate TAG and WE has been reported for only some genera (Alvarez and Steinbüchel 2002). The amount, composition and structure of bacterial lipids are dependent on several factors, including the bacterial species itself, the carbon source used, the time of cultivation and the amount of carbon and nitrogen present in the culture medium (Packter and Olukoshi 1995; Alvarez et al. 1997a, 1997b; Wältermann et al. 2005). Among several TAG accumulating genera, the genus *Rhodococcus* is one of the most promising, because some strains can accumulate more than 20 % of their biomass as TAG, being considered oleaginous bacteria. Members of this genus can be found in several types of natural environments, from arid and tropical soils to cold ecosystems and marine sediments (Whyte et al. 1999; Heald et al. 2001; Luz et al. 2004; Peng et al. 2008). Additionally, *Rhodococcus* can produce and accumulate TAG from several types of substrates under nitrogen-limiting conditions, including defined carbons sources like sugars, organic acids or hydrocarbons (Alvarez et al. 1996, 1997a, 2000; Silva et al. 2010) but also complex carbon sources present in industrial wastes (Voss and Steinbuchel 2001; Gouda et al. 2008), revealing a remarkable versatility in terms of substrate degradation.

Within the last decade, reports of new TAG accumulating species of *Rhodocococcus* have considerably increased, for example *R. ruber*, *R. fascians*, *R. erythropolis*, *R. jostii*, *R. aetherivorans* IAR1, *R. sp* 602 and *R. sp*. A5 (Alvarez et al. 1997a, 2000; Kalscheuer et al. 2001; Alvarez 2003; Hernandez et al. 2008; Hori et al. 2009; Silva et al. 2010; Bequer Urbano et al. 2013). *Rhodococcus opacus* PD630 is the best studied bacterium concerning TAG production and accumulation. This bacterium has the ability to accumulate significant amounts of lipids, namely 76 and 87 % (w/w) of the cellular dry weight (CDW), when grown on gluconate and olive oil, respectively (Alvarez et al. 1996; Voss and Steinbüchel 2001). Additionally, it can accumulate TAG under cultivation on other carbon sources, such as alkanes, acetate, glucose, propionate, among others (Alvarez et al. 1996; Wältermann et al. 2000; Alvarez and Steinbüchel 2010). For these reasons, *R. opacus* PD630 is considered a model bacterium. However, to develop sustainable lipids producing processes using *Rhodococcus* sp. and to increase the knowledge of TAG metabolism and physiology in this species it is mandatory to get further insights on lipid accumulation and also to identify new *Rhodococcus* strains possessing this feature. *R. opacus* B4 was isolated from a gasoline-contaminated soil in Japan. This bacterium is highly tolerant to several organic solvents, can use benzene as a sole source of carbon and energy and metabolizes aromatic and aliphatic hydrocarbons (Na et al. 2005; Yamashita et al. 2007; Sameshima et al. 2008). Moreover, it has the capacity to stabilize water–oil emulsions, which can be important in bioremediation processes (Honda et al. 2008; Hamada et al. 2009). However, the ability to produce and accumulate lipid storage compounds was never reported. In the present work, the ability of *R. opacus* B4 to produce and accumulate lipid storage compounds was investigated, using different carbon sources and accumulation period lengths. *R. opacus* PD630 was used under the same cultivation conditions as a comparative well characterized bacterium.

## Materials and methods

### Bacterial strains, media and cultivation conditions

#### Strains and media

*Rhodococcus opacus* PD630 (DSM 44193) and *Rhodococcus opacus* B4 (NBRC 108011) were purchased from the Deutsche Sammlung von Mikroorganismen und Zellkulturen (DSMZ) and Biological Resource Center, NITE (NBRC), respectively. *R. opacus* PD630 was isolated from a soil sample collected at a gas-works plant in Germany (Alvarez et al. 1996) and *R. opacus* B4 was isolated from an oil sample taken from a gasoline contaminated roadside in Hiroshima, Japan (Na et al. 2005).

The culture media used for maintenance and growth of bacterial strains were 802 medium containing (g L^−1^) 10.0 polypeptone; 2.0 yeast extract and 1.0 MgSO_4_·7H_2_O and mineral salts (MS) medium according to (Schlegel et al. 1961. Glucose (40 g L^−1^), sodium acetate (10 g L^−1^) or hexadecane (1 g L^−1^) were used as carbon and energy sources. For solid media, 1.5 % agar was added to the MS and 802 media. The cells were grown at 30 °C and 150 rpm under the conditions described below.

#### Preparation of seed cultures

Cells from a single colony of *R. opacus* B4 and *R. opacus* PD630, grown on 802 medium agar plates at 30 °C for 4 days, were separately inoculated in 50 mL of 802 medium in 250 mL flasks. The seed cultures were incubated on a rotary shaker (150 rpm) at 30 °C until the middle of the exponential growth phase was reached (48 h for *R. opacus* B4 and 24 h for *R. opacus* PD630). Growth of the seed cultures was determined by measuring optical density at 600 nm (nm) wavelength with a spectrophotometer (U-1500 Hitachi, Tokyo, Japan).

#### Growth and lipid accumulation experiments

The experiments were divided in two cultivation stages. In stage I, culture conditions promoted high cell densities (growth conditions) while in stage II neutral lipid accumulation was promoted (storage conditions). MS medium supplemented with glucose (4 %, w/v), sodium acetate (0.6 %, w/v) or hexadecane (0.3 %, w/v) as sole carbon sources was used for cultivation.

The two-stage experiments were carried out under sterile conditions in duplicate using 250 mL conical flasks containing 50 mL of defined medium and incubated on a rotary shaker (250 rpm) at 30 °C. In stage I, cells of seed cultures were harvested, washed with sterile NaCl solution (0.9 %, w/v), and suspended in fresh MS medium. Cells were used to inoculate flasks to an optical density (OD) at 600 nm of 0.1. Cultures of both strains were cultivated in MS medium at a molar carbon to nitrogen ratio of 68 for glucose (C/N = 68), 8 for acetate (C/N = 8) and 4 for hexadecane (C/N = 4). Cells were grown until the middle of the exponential growth phase, namely 32, 50 and 150 h for glucose, acetate and hexadecane, respectively.

For stage II, cells from stage I were collected by centrifugation (4 °C, 10 min., 10,000*g*), washed twice with sterile NaCl solution (0.9 % w/w) and transferred to fresh MS medium with lower nitrogen concentration, with a C/N ratio of 300 for all carbon sources, in order to promote neutral lipid accumulation. After 24 and 72 h of cultivation, cells were harvested, washed and kept at −80 °C until further lyophilization.

### Substrate consumption

#### Glucose and acetate

Samples from the culture medium of both strains were collected in stage II at 0, 24 and 72 h to analyze substrate concentration. Glucose and acetate were measured by high-performance liquid chromatography (Jasco, Japan) using a Chrompack column (6.5 × 30 mm^2^). The mobile phase used was sulfuric acid (0.01 N) at a flow rate of 0.6 mL min^−1^. The column temperature was set at 60 °C. Detection of compounds was performed by using an UV detector at 210 nm for acetate and a refraction index (RI) detector for glucose.

#### Hexadecane

For hexadecane analysis, total content of the cultivation flask (50 mL of culture medium) was acidified with HCl 8 M to pH 2 and stored at 4 °C (for no longer than 14 days) until extraction. Hexadecane present in the culture medium was extracted using a liquid–liquid extraction procedure. Before extraction, tetradecane was added as a surrogate to evaluate the efficiency of the extraction process. The extraction was serially performed three times with 15 mL of hexane. Between the serial extractions, the funnels were vigorously shaken for 2 min. After 10 to 30 min rest (depending on the sample), the organic layer was collected.

The extract was passed through a Sep-Pak Florisil 6 cc column (Waters, USA) using a Supelco^®^ vacuum Manifold system. The column was previously preconditioned with 5 mL n-hexane. In the end, an additional 5 mL n-hexane was added to the column to ensure that all hexadecane was eluted. Then, sodium thiosulfate was added to remove residual aqueous residues. The extracts were kept in glass flasks in fume hood until dryness, reconstituted in 1 mL n-hexane and stored at −20 °C until analysis. Hexadecane concentration was determined using a gas chromatograph with a flame ionization detector (GC-FID) (GC Varian^®^ star 3400 CX, USA). Undecane (C11) was used as internal standard and was added to the samples before GC analysis. The column used was the model VF-1 ms (Agilent, USA) 30 m length long × 0.025 mm internal diameter, made from fused silica coated with dimethylpolysiloxane as stationary phase. Helium was used as carrier gas at 1 mL min^−1^. The temperature of the detector and the injector were set at 300 and 285 °C, respectively. The column’s temperature was maintained at 60 °C for 1 min and then programmed up to 290 °C at a rate of 8 °C min^−1^.

### Extraction and analysis of cellular lipids

#### Lipid extraction

Lipids were extracted according to Folch method (Folch et al. 1957), with some modifications. 15 mg of lyophilized cells were extracted with 3 mL of a mixture of chloroform/methanol (2:1, v/v) (Wältermann et al. 2000). The mixture was incubated at room temperature with shaking for 2 h. The crude extract was filtered through a Pasteur pipette packed with glass wool (1.5 cm packing) to separate the cell debris and salts and afterwards evaporated to dryness.

#### Neutral lipid detection by TLC

Analysis of lipid content in *R. opacus* B4 and *R. opacus* PD630 was performed by thin-layer chromatography (TLC). Lipid extracts were dissolved in chloroform/methanol (2:1, v/v). The extracts were applied on 10 × 10 cm glass DC-Fertigplatten plates, precoated with 0.25 mm silica gel 60 with fluorescent indicator UV254 (60F254 silica gel plates-Merck, Darmstadt, Germany). The TLC chromatogram was performed applying the following developing solvent system: hexane/diethyl ether/acetic acid (80:20:1, v/v/v) as mobile phase for TAG analysis (Wältermann et al. 2000).

Neutral lipids were visualized on plates after brief exposure to iodine vapor. Olive oil, oleic acid, and oleyl oleate were used as standard substances for TAG, FA and WE, respectively.

#### TAG quantification

After evaporation of iodine, TAG bands were scraped from the TLC plates and transfer to a Pasteur pipet containing glass wool. TAG fraction was eluted from Silica Gel by adding three times 1 mL chloroform and transferred to a previously weighted glass flask. Chloroform was evaporated in the fume hood and TAG amounts were determined gravimetrically (Santala et al. 2011).

#### Analysis of cellular fatty acid content and composition

Approximately 15 mg of whole lyophilized cells or 5 mg of TAG fraction obtained from TLC (depending on the strain) were submitted to methanolysis in the presence of 15 % (v/v) sulfuric acid (Brandl et al. 1988; Timm et al. 1990). Briefly, samples were added to Teflon screw-capped glass test tubes, mixed with 1.5 mL chloroform to which 3 ml of a 85:15 methanol/sulphuric acid solution was added. The tubes were heated at 100 °C for 3.5 h. Subsequently, 1 mL of ultra-pure water was added, and the samples were vortexed for 1 min, and after phase separation (approximately 30 min), the organic phase was collected, containing the fatty acid methyl esters. Heptanoic acid (C7:0) and pentadecanoic acid (C15:0) were used as internal standards, added to the samples before methanolysis takes place (1.5 mL). Methyl esters were quantified in a GC-FID (Varian 3800). FAMEs were separated on a CP-Sil 52 CB 30 m × 0.32 mm × 0.25 µm capillary column (Teknokroma, TR-WAX). One microlitre portion of the organic phase was analyzed using a splitless mode. Helium was used as carrier gas at a flow rate of 1.0 mL min^−1^. A temperature program was established for an efficient separation of the methyl esters. Initial oven temperature was set at 50 °C for 2 min with an increase of 10 °C min^−1^ to a final temperature of 225 °C. Injector and detector temperatures were set at 220 and 250 °C, respectively. FA were identified by comparison of the respective retention factor values (Rf) of standard fatty acid methyl esters.

### Statistical analysis

Significant differences between biological samples, cultivated on different carbon sources and on different accumulation periods, were evaluated using two factor analyses of variances (ANOVA), using SPSS 22.0.0 statistical software. Statistical significance was established at the p < 0.05 level.

## Results

### Biomass production and substrate degradation

In both strains, maximum biomass density was obtained with glucose, whereas the minimum was achieved with hexadecane as carbon source (Table 1). A significant increase in biomass production by increasing the incubation period (from 24 to 72 h) was observed when cells of *R. opacus* B4 were cultivated on glucose, reaching the highest value observed for this strain, namely 3.3 ± 0.01 g L^−1^ (p < 0.05). On the other hand, the effect of incubation time in *R. opacus* PD630 was not observed, although it was able to reach higher cell densities than *R. opacus* B4 at 24 and 72 h, namely 1.5- and 1.3-fold more (p < 0.05).

**Table 1:** Cell dry matter (CDW), triacylglycerol (TAG) content, % of substrate degradation and TAG yields in cells of *R. opacus* B4 and *R. opacus* PD630

Strain	Carbon source	Time (h)	CDW (g L^−1^)	TAG (g g^−1^ CDW)	Substrate consumed (%)	TAG yield (g g^−1^ substrate consumed)
*R. opacus* B4	Glucose	0	2.2^a^	0.044^a^		
		24	2.5 ± 0.04	0.230 ± 0.045	8.1 ± 4.3	0.203 ± 0.068
		72	3.3 ± 0.01	0.217 ± 0.014	37.4 ± 5.6	0.054 ± 0.015
	Acetate	0	0.45^a^	0.081^a^		
		24	1.1 ± 0.1	0.252 ± 0.040	97.3 ± 0.5	0.054 ± 0.005
		72	0.724 ± 0.017	0.117 ± 0.061	100.0 ± 0.0	0.016 ± 0.002
	Hexadecane	0	0.398^a^	0.079^a^		
		24	0.459 ± 0.134	0.186 ± 0.021	70.5 ± 5.1	0.147 ± 0.069
		72	0.837 ± 0.281	0.170 ± 0.034	85.2 ± 17.7	0.194 ± 0.112
*R. opacus* PD630	Glucose	0	2.4^a^	0.111^a^		
		24	3.9 ± 0.4	0.287 ± 0.003	22.8 ± 0.8	0.076 ± 0.001
		72	4.1 ± 0.5	0.384 ± 0.051	26.5 ± 5.8	0.125 ± 0.024
	Acetate	0	0.620^a^	0.169^a^		
		24	1.1 ± 0.01	0.246 ± 0.015	81.1 ± 10.4	0.054 ± 0.003
		72	0.726 ± 0.040	0.444 ± 0.179	100.0 ± 0.0	0.059 ± 0.033
	Hexadecane	0	0.250^a^	0.063^a^		
		24	0.321 ± 0.168	0.141 ± 0.013	52.6 ± 9.8	0.070 ± 0.049
		72	0.468 ± 0.062	0.092 ± 0.008	94.5 ± 4.2	0.037 ± 0.004

Cells were cultivated in different carbon sources under nitrogen limiting conditions (C/N = 300) during 24 and 72 h

The means are the result of at least two independent experiments, ± standard deviation (s.d.)

^a^Corresponds to one replicate

*R. opacus* B4 presented higher growth than *R. opacus* PD630 using hexadecane as carbon and energy source. When incubation time was increased to 72 h, *R. opacus* B4 doubled biomass concentration to a density of 0.837 ± 0.281 g L^−1^ while *R. opacus* PD630 presented similar values in both incubation times, but about half of the obtained for *R. opacus* B4 (p < 0.05). Acetate had a different behavior when compared to the other carbon sources. For both strains, there was a 35 % decrease in CDW from 24 to 72 h incubation period (p < 0.05).

Regarding substrate consumption, there was a general increase with incubation time for both strains and for all carbon sources (Table 1). Cells of *R. opacus* B4 consumed less glucose in the first 24 h (8.1 ± 4.1 %) when compared to *R. opacus* PD630 (22.8 ± 0.8 %) (p < 0.05). When cultivated on hexadecane, *R. opacus* B4 degraded more substrate at 24 h (70.5 ± 5.1 %) than *R. opacus* PD630 (52.6 ± 9.6 %) (p < 0.05). Acetate was almost entirely consumed by both strains during the first 24 h incubation time (97.3 % in *R.opacus* B4 and 81.1 % in *R. opacus* PD630), reaching full depletion at 72 h.

### Neutral lipids profile, TAG levels and yields

**Fig. 1:**
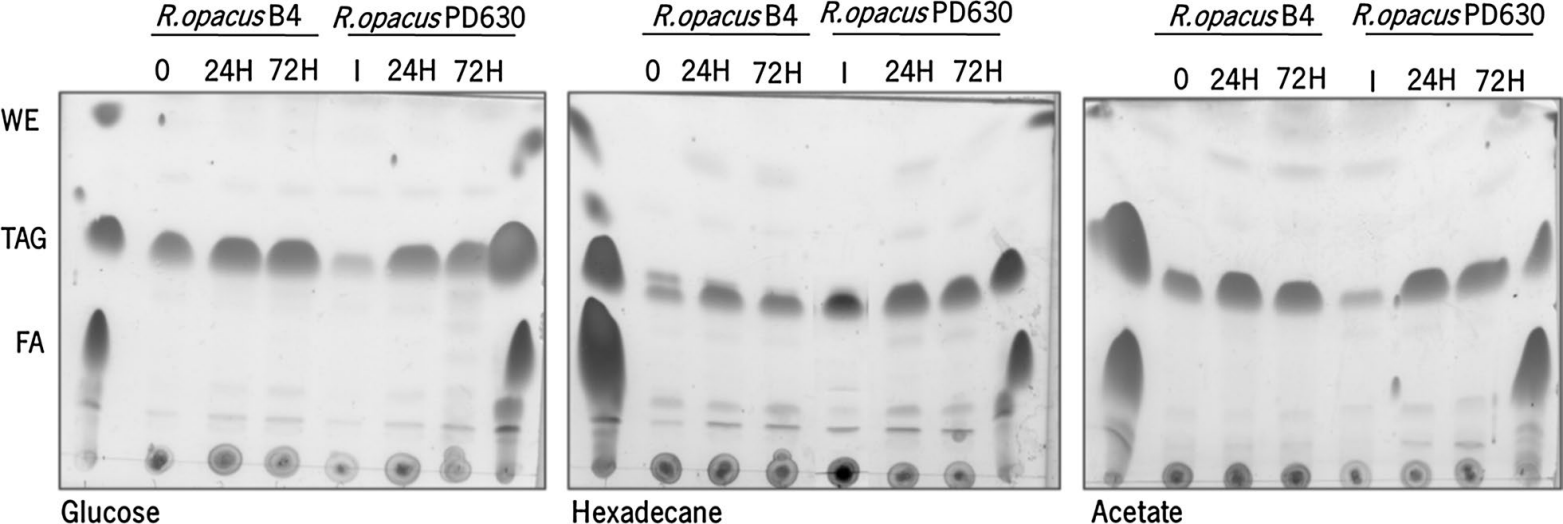
TLC analysis of storage lipid accumulation in *R. opacus* B4 and *R. opacus* PD630. Cells were cultivated on 4 % glucose (w/w), 0.6 % acetate (w/w) and 0.1 % hexadecane (w/w). After growth, cells were transferred to MS medium with a molar ratio of C/N 300 during 24 and 72 h. *TAG* triacylglycerols; *WE* wax esters; *FA* fatty acids

The patterns of neutral lipids produced by *R. opacus* B4 and by *R. opacus* PD630 are illustrated in Fig. 1. The most intense TLC band observed in all tested conditions corresponds to TAG. Furthermore, FA and WE were also present but in minor amounts. Three additional faint bands of unknown identity were visualized: located below FA, immediately below TAG bands and between TAG and WE.

The highest and the lowest TAG levels were obtained by *R. opacus* PD630 cultivated for 72 h in acetate and hexadecane, respectively (Table 1). In glucose, differences in cultivation length did not affect significantly TAG levels in *R. opacus* B4, but an increase of 34 % was observed in *R. opacus* PD630. *R. opacus* PD630 was able to accumulate more TAG than *R. opacus* B4 especially at 72 h (p < 0.05). On acetate, *R. opacus* B4 suffered a decrease of more than 50 % of TAG levels from 24 to 72 h. On the other hand, *R. opacus* PD630 increased TAG production with incubation time, achieving a maximum of 0.444 g g^−1^ CDW at 72 h (p < 0.05), which was 3.8-fold higher (p < 0.05) than the one obtained with *R. opacus* B4.

For hexadecane, the amount of TAG was not affected by incubation time in *R. opacus* B4 whereas *R. opacus* PD630 had a 35 % decrease in TAG content (p < 0.05).

Regarding TAG yields on substrate, maximum and minimum values were achieved by *R. opacus* B4 grown on glucose for 24 h (0.203 g g^−1^ substrate consumed) and acetate during 72 h (0.016 g g^−1^ substrate consumed), respectively (Table 1). For *R. opacus* PD630 the highest yield was obtained at 72 h using glucose as carbon source, whereas hexadecane was the most unsuitable carbon source, resulting in the lowest yield. As for the TAG content, increasing the accumulation period length from 24 to 72 h also influenced TAG yield in *R. opacus* B4 growing on acetate, resulting in a 3.4-fold decrease. TAG yield, in *R. opacus* PD630 was not affected under the same conditions.

With hexadecane, *R. opacus* B4 presented similar yields for both incubation periods, 2 and 5 fold higher yields than *R. opacus* PD630 (p < 0.05) for 24 and 72 h respectively. In *R. opacus* PD630 a 47 % decrease was observed after 72 h.

### Fatty acid composition of biomass and TAG produced

Fatty acid composition of intact cells was not influenced by incubation time but drastically affected by the substrate used (Table 2). With glucose and acetate, and for both strains, fatty acids varied from 14 to 18 carbon atoms, being hexadecanoic acid (C16:0) the predominant one. In *R. opacus* B4 the fraction of saturated fatty acids was about 65 % and odd numbered fatty acids accounted for about 40 and 50 % for glucose and acetate, respectively, whereas in *R. opacus* PD630 these fractions were 35 and 55 %, respectively.

**Table 2:** Total fatty acid (FA) content and composition as % of total fatty acids (g g^−1^) in cells of *R. opacus* B4 and *R. opacus* PD630

Strain	Substrate	Time (h)	FA (g g-^1^ CDW)	Relative proportion of fatty acids [%, w/w]								
				C10:0	C12:0	C14:0	C16:0	C16:1	C17:0	C17:1	C18:0	C18:1
*R. opacus* B4	Glucose	0	0.364^a^	–	–	1.7	33.6	5.7	36.6	8.2	4.4	9.9
		24	0.528 ± 0.049	–	–	2.0 ± 0.16	38.5 ± 0.74	5.0 ± 0.44	22.9 ± 0.58	15.0 ± 0.09	2.8 ± 0.34	13.7 ± 0.34
		72	0.613 ± 0.051	–	–	1.6 ± 0.04	35.6 ± 0.19	4.5 ± 0.01	22.4 ± 1.21	16.5 ± 0.47	3.8 ± 0.27	15.6 ± 0.27
	Acetate	0	0.235^a^	–	–	2.2	44.5	3.8	14.8	7.6	14.2	12.2
		24	0.664 ± 0.013	–	–	1.4. ± 0.04	33.4 ± 0.43	4.1 ± 0.08	26.5 ± 1.00	20.3 ± 0.94	0.91 ± 0.15	13.4 ± 1.06
		72	0.517 ± 0.014	–	–	1.3 ± 0.03	29.9 ± 0.79	4.4 ± 0.05	25.5 ± 0.63	22.8 ± 0.56	–	16.1 ± 0.84
	Hexadecane	0	0.087^a^	–	–	1.1	54.0	37.2	–	–	–	4.2
		24	0.273 ± 0.088	–	–	4.3 ± 0.55	48.3 ± 8.9	45.8 ± 11.71	–	–	–	3.2 ± 0.72
		72	0.285 ± 0.037	4.8 ± 0.497	0.6 ± 0.009	4.2 ± 0.15	54.4 ± 0.18	31.5 ± 3.41	–	–	2.6 ± 0.49	3.1 ± 1.05
*R. opacus* PD630	Glucose	0	0.525^a^	–	–	1.6	38.1	9.6	22.4	16.2	3.8	8.3
		24	0.507 ± 0.013	–	–	1.4 ± 0.04	37.6 ± 0.67	11.6 ± 0.45	12.8 ± 0.31	19.5 ± 0.39	2.3 ± 0.43	14.8 ± 0.41
		72	0.580 ± 0.014	–	–	1.3 ± 0.08	33.3 ± 0.11	11.8 ± 0.31	15.9 ± 0.04	21.4 ± 0.20	2.4 ± 0.12	14.8 ± 0.17
	Acetate	0	0.238^a^	–	–	1.9	39.5	17.6	9.6	19.3	–	12.1
		24	0.505 ± 0.006	–	–	1.6 ± 0.41	36.8 ± 3.45	10.7 ± 1.64	15.4 ± 1.19	22.1 ± 3.52	6.8 ± 0.97	10.0 ± 1.46
		72	0.449 ± 0.001	–	–	1.3 ± 0.12	30.5 ± 0.87	12.3 ± 0.12	15.9 ± 0.04	28.1 ± 0.78	–	11.8 ± 0.02
	Hexadecane	0	0.116^a^	–	–	3.2	35.3	59.1	–	–	–	2.4
		24	0.218 ± 0.003	–	–	4.1 ± 0.38	53.6 ± 4.21	42.4 ± 038	–	–	–	–
		72	0.254 ± 0.041	–	0.5 ± 0.009	3.7 ± 0.06	51.7 ± 1.27	48.9 ± 1.47	–	–	–	2.0 ± 0.37

Cells were cultivated in different carbon sources under nitrogen limiting conditions (C/N = 300) during 24 and 72 h

The means are the result of at least two independent experiments, ± s.d

^a^Corresponds to one replicate

– Not detected or <0.5 %

For hexadecane, fatty acids with lower carbon chains (C10–C16) were mainly detected, being hexadecanoic (C16:0) and hexadecenoic (C16:1) acids the predominant ones. In both strains, even-numbered fatty acids were 100 %.

Comparing both strains for glucose and acetate, the main differences were obtained for the percentage of C16:1 that was approximately two-fold higher in *R. opacus* PD630 than in *R. opacus* B4, and of C17:0, that corresponded to around 23 to 27 % of the fatty acids present in *R. opacus* B4 and around 13 to 16 % in *R. opacus* PD630 (p < 0.05). With hexadecane, decanoic acid (C10:0), dodecanoic acid (C12:0) and stearic acid (C18:0) were only detected in *R. opacus* B4.

The fatty acid patterns of produced TAG fraction showed some important differences, depending on the strain, accumulation period length and carbon source used (Table 3). In both strains cultivated on glucose and acetate, the most dominant fatty acids were C16:0 (35 to 55 %) and C17:0, ranging between 15 and 40 % for glucose, and between 35 and 50 % for acetate. For hexadecane, the predominant fatty acids were C10:0 (almost 30 %) and C16:0 (55 %) in *R. opacus* B4, and C16:0 (85 %) in *R. opacus* PD630. In *R. opacus* B4, the fraction of saturated fatty acids was similar for all carbon sources, reaching 90 to 96 %. Even-numbered fatty acids were 55 to 65 % for glucose and acetate, and 100 % for hexadecane. In *R. opacus* PD630, with hexadecane, no odd-numbered fatty acids were detected either, and 60 to 70 % of even-numbered fatty acids were found with glucose and acetate. In both strains cultivated on glucose, there was a transition between saturated and unsaturated fatty acids from 24 to 72 h incubation length. C10:0 and C12:0 were only detected in *R. opacus* B4. TAG from *R. opacus* B4 cultivated on glucose and acetate had a higher percentage of C17:0 (39 and 48 %, respectively) than from *R. opacus* PD630 (16 and 38 %). In contrast, in *R. opacus* PD630 TAG contained higher percentages of C14:0 (13 %); C16:0 (86 %) and C16:1 (39 %) when compared to those of *R. opacus* B4 in hexadecane.

**Table 3:** Fatty acid composition as  % of total fatty acids (g g^−1^) in TAG fraction of *R. opacus* B4 and *R. opacus* PD630 cultivated in different carbon sources under nitrogen limiting conditions (C/N = 300) during 24 and 72 h

Strain	Substrate	Time (h)	Relative proportion of fatty acids [%, w/w]								
			C10:0	C12:0	C14:0	C16:0	C16:1	C17:0	C17:1	C18:0	C18:1
*R. opacus* B4	Glucose	0	–	6.7	7.6	41.7	–	44.1	–	–	–
		24	–	1.2 ± 0.09	3.2 ± 0.69	49.7 ± 0.12	–	38.8 ± 4.29	4.2 ± 1.46	3.3 ± 0.46	4.0 ± 1.42
		72	–	1.4 ± 0.42	3.1 ± 0.03	38.3 ± 1.13	3.2 ± 0.21	24.8 ± 0.32	14.4 ± 0.37	2.0 ± 0.01	13.2 ± 0.26
	Acetate	0	–	15.8	16.5	26.3	16.7	–	–	–	28.4
		24	–	–	2.9 ± 1.17	48.6 ± 1.75	–	47.6 ± 1.28	–	–	3.4 ± 1.83
		72	–	–	5.3 ± 0.57	41.1 ± 4.02	–	48.1 ± 3.42	–	–	3.9 ± 0.16
	Hexadecane	0	–	1.1	5.4	70.2	16.2	–	–	–	7.8
		24	27.4 ± 0.8	1.3 ± 0.08	5.7 ± 0.17	54.0 ± 1.12	9.5 ± 0.29	–	–	–	2.0 ± 0.21
		72	28.9 ± 1.36	1.1 ± 0.51	6.1 ± 0.41	55.0 ± 5.1	6.8 ± 0.14	–	–	–	2.1 ± 0.68
*R. opacus* PD630	Glucose	0	–	–	4.2	48.9	2.9	31.4	5.2	5.1	2.0
		24	–	–	2.8 ± 0.52	48.4 ± 4.43	6.1 ± 2.7	18.7 ± 1.25	12.7 ± 0.43	3.1 ± 0.09	8.2 ± 3.08
		72	–	–	2.0 ± 0.04	35.3 ± 2.73	6.3 ± 2.44	16.3 ± 1.00	11.9 ± 2.31	2.5 ± 0.28	25.7 ± 5.6
	Acetate	0	–	–	7.3	68.6	–	24.3	–	–	–
		24	–	–	3.8 ± 0.19	55.1 ± 6.72	–	36.7 ± 1.58	8.6 ± 1.16	–	–
		72	–	–	4.5 ± 0.47	55.9 ± 2.92	–	36.1 ± 1.97	6.5 ± 1.4	–	–
	Hexadecane	0	–	–	28.4	69.2	–	–	–	–	–
		24	–	–	13.3 ± 3.91	86.7 ± 3.91	–	–	–	–	–
		72	–	–	7.3 ± 2.86	70.4 ± 7.79	19.8 ± 7.12	–	–	–	2.4 ± 0.39

The means are the result of at least two independent experiments, ± s.d

^a^Corresponds to one replicate

– Not detected or <0.5 %

## Discussion

This study contributes to increase the knowledge of TAG metabolism and physiology in *Rhodococcus* sp., but for practical applications, it is important to analyze global volumetric TAG productions and to extract objective conclusion with interest for industrial and environmental biotechnological applications.

*R. opacus* B4 showed a TAG production capability from hexadecane 3.3 fold higher than the volumetric production obtained by *R. opacus* PD630 (0.14 versus 0.043 g L^−1^ at 72 h cultivation time). This is relevant as this strain was never reported to accumulate TAG although it was reported to be able to transform and degrade several types of hydrocarbons and to stabilize water–oil phases (Na et al. 2005; Yamashita et al. 2007; Honda et al. 2008; Sameshima et al. 2008). For acetate, both strains showed similar maximal TAG volumetric productions (about 0.3 g L^−1^). In *R. opacus* B4 acetate consumption was faster, and the exhaustion of the external carbon source likely induced the decrease observed in TAG levels for the longest incubation period (72 h), due to its mobilization for cell maintenance. TAG’s use as internal carbon and energy source has been also reported by Alvarez et al. (2000) in *R. opacus* PD630 and in *R. ruber* NCIMB 40126 cultivated on gluconate and glucose, after depletion of these carbons sources. With glucose, *R. opacus* PD630 showed about 2 fold the production obtained for *R. opacus* B4 in the same incubation periods (1.1 and 1.6 g L^−1^).

The type of carbon source is one of the most determinant factor influencing fatty acid content and composition in bacteria. *R. opacus* B4 presented a higher level of total fatty acids (intact cells) when cultivated on glucose or acetate than in hexadecane, and exhibited higher percentages than *R. opacus* PD630.

Taking into account the results obtained, *R. opacus* B4 can be considered an oleaginous bacterium, since it can accumulate more than 20 % of biomass as lipids. There are several studies in literature reporting fatty acid accumulation in several *Rhodococcus* species using these substrates, ranging between 3.8 and 48.4 % for glucose (Alvarez et al. 1997a; Shields-Menard et al. 2015), 21 and 31 % for acetate (Hernandez et al. 2008; Hori et al. 2009) and between 8.1 and 43.4 % for hexadecane (Alvarez 2003). Table 4 gives an overview of total (whole cell) fatty acid composition reported for different *Rhodococcus* highlighting the FA production potential of *R. opacus* B4 herein reported for the first time.

**Table 4:** Comparison of total fatty acids production between different lipid accumulating *Rhodococcus* using sugar, organic acid and hydrocarbon defined substrates

Strain	Substrate	Incubation time (h)	FA (g g^−1^ CDW^a^)	FA (g L^−1^)	Reference
*R. opacus* B4	Glucose (40 g L^−1^)	24	0.528	0.600	This study^b^
		72	0.613	0.700	
	Acetate (6 g L^−1^)	24	0.664	0.300	
	Hexadecane (1 g L^−1^)	24	0.273	0.090	
*R. opacus* PD630	Glucose (40 g L^−1^)	24	0.507	1.1	
		72	0.580	1.6	
	Acetate (6 g L^−1^)	24	0.505	0.270	
	Hexadecane (1 g L^−1^)	24	0.218	0.045	
	Glucose (16 g L^−1^)^c^	72	0.516	2.87	Kurosawa and Sinskey (2013)
	Glucose (240 g L^−1^)	147	0.380	25.2	Kurosawa et al. (2010)
	Glucose (10 g L^−1^)	12	0.198		Kosa and Ragauskas (2012)
	Glucose (40 g L^−1^)	96	0.412		MacEachran et al. (2010)
	Glucose (40 g L^−1^)	120	0.378		MacEachran and Sinskey (2013)
	Acetate (6 g L^−1^)	24	0.310		Alvarez et al. (1997a)
	Hexadecane (1 g L^−1^)	48	0.380		
*R. opacus* DSM1069	Glucose (10 g L^−1^)	12	0.216	0.244	Wei et al. (2015)
		120	0.179		Kosa and Ragauskas (2012
*R. ruber* NCIMB 40126	Glucose (10 g L^−1^)	24	0.190		Alvarez et al. (1997a)
		84	0.380		Alvarez et al. (2000)
	Hexadecane (1 g L^−1^)	24	0.260		
*Rhodococcus* sp. A5	Glucose (10 g L^−1^)	72	0.176		Bequer Urbano et al. (2013)
	Hexadecane (1 g L^−1^)	72	0.283		
*R. fascians* 123	Glucose (10 g L^−1^)	24	0.038		Alvarez et al. (1997a)
	Hexadecane (1 g L^−1^)	24	0.181		
		72	0.129		Alvarez (2003)
*R. erythropolis* 17		72	0.434		
		24	0.176		Alvarez et al. (1997a)
*Rhodococcus* sp. 20		72	0.081		Alvarez (2003)
*R. aetherivorans* IAR1	Acetate (9 g L^−1^)	80	0.240		Hori et al. (2009)
	Toluene (0.5 g L^−1^)	50	0.240		
*Rhodococcus* sp. *602*	Hexadecane (1 g L^−1^)	48	0.223		Silva et al. (2010)
*R. rhodochrous*	Glucose (20 g L^−1^)	168	0.430	3.030	Shields-Menard et al. (2015)
*R. jostii* RHA1	Glucose (nr)	nr	0.484		Hernandez et al. (2008)
	Acetate (nr)	nr	0.212		
	Hexadecane (nr)	nr	0.304		

^a^Cellular dry weight

^b^Conditions with possible substrate limitation are not included

^c^Engineered *R. opacus* PD630 strain to utilize xylose

*nr* not reported

In general, the results obtained for fatty acid content in the produced TAG fraction are in agreement with the ones obtained for intact cells and a high variability of fatty acids was obtained, influenced by the type of carbon source used. When cells were cultivated on hexadecane, only even-numbered fatty acids were detected in the produced TAG, being C16:0 the dominant one. This suggests that the fatty acids produced were directly related to the chain length of the carbon source and to ß-oxidation pathway, where acetate (C2) is repeatedly removed, and agrees with other works (Alvarez et al. 1996; Alvarez et al. 1997a; Gouda et al. 2008; Hernandez et al. 2008; Silva et al. 2010). TAG composition can be very diverse among different species or even among strains of the same species in *Rhodococcus* genus, depending on genetic and metabolic machinery present in each organism (Alvarez et al. 2013). In *R. opacus* B4 shorter even-numbered fatty acids were detected in TAG, likely due to the presence of several DGAT enzymes that can have different substrate specificities in this strain compared to strain PD630 (Villalba et al. 2013). In the case of acetate and glucose, C16:0 and C17:0 were the dominant fatty acids, with higher percentage in *R. opacus* B4. These substrates can be converted into acetyl-CoA which is the precursor of de novo fatty acid synthesis pathway. Presence of odd-numbered fatty acids can result from the presence of an effective methylmalonyl-CoA production through succinyl-CoA (derived from TCA cycle), resulting in the formation of propionyl-coA, a 3-carbon molecule, which is a precursor for the synthesis of odd-numbered fatty acids. Several *Rhodococcus* species exhibited similar pattern (Alvarez et al. 1996, 1997a; Alvarez 2003; Hernandez et al. 2008; Holder et al. 2011).

*Rhodococcus opacus* B4 produced a high content of lipids, mainly composed by fatty acids ranging between C16 and C18 and revealed a high proportion of saturated acids over unsaturated ones. These properties are identical to the ones of traditional vegetable oils currently applied in biodiesel industry, namely canola, palm, sunflower, soybean and rapeseed oils (El-Hawa et al. 2004; Moser 2008). This suggests that *R. opacus* B4 can be exploited for high quality biodiesel-compatible fatty acids production (Ramos et al. 2009).

Furthermore, the production of TAG containing shorter fatty acids, namely decanoic acid (C10:0) by *R. opacus* B4 is an interesting feature. Decanoic acid (capric acid) is a saturated fatty acid that can be used in different types of industrial and commercial applications mainly due to its different chemical properties. This medium chain fatty acid is used in the production of creams and lotions in the personal care industry (Zielinska and Nowak 2014), as well as in the production of artificial flavors and aromas in the food and beverage industry (Saerens et al. 2008). In the pharmaceutical industry it is used as an antiviral and antimicrobial agent and also as a vasodilator (German and Dillard 2014). Additionally, it is applied in the textile industry and dyes production and is also useful in the production of synthetic rubber. It can be used as a plasticizer in the manufacture of a wide range of plastics and lubricating grease. In nature, capric acid can only be found in coconut oil, palm kernel oil and in goat, cow and human milks. Growing demand for those products around the world will require an increasing demand for capric acid in the next years, according to Global Capric Acid Market for Artificial Fruit Flavors and Perfumes, Pharmaceuticals and Chemical Syntheses (Lubricants, Greases, Rubber, Dyes, Plastics, etc.) 2014—2020 report (2016). Scarcity in availability of raw materials will be a relevant challenge to the development of capric acid based industry. Therefore, *R. opacus* B4 can be considered as a valuable alternative to the bioprocesses based on edible feedstocks.

Although focus on current research has been much directed to increase TAG production in *R opacus* PD630, such as running the process at a higher scale (Kurosawa et al. 2010), as well as to perform metabolic engineering studies (Hetzler and Steinbüchel 2013; Kurosawa et al. 2013, 2015a, 2015b), new strains of *Rhodococcusopacus* should be also considered and explored for similar abilities. In particular the strain studied in this work, *R. opacus* B4, shows relevant interest in TAG production from liquid hydrocarbons, herein demonstrated with hexadecane as a model alkane. Combining TAG production with hydrocarbons degradation is a powerful strategy to achieve environmental remediation while producing added value compounds.

In conclusion, the obtained results contribute to increase the knowledge of TAG metabolism and physiology in *Rhodococcus* sp. and open new perspectives for the use of *R. opacus* B4 to produce triacylglycerol with fatty acids composition relevant for industrial purposes, in particular using abundant recalcitrant residues like alkane-based wastewater as cheap raw-material.

## Abbreviations

CDW: cellular dry weight
FA: fatty acids
FID: flame ionization detector
MS: mineral salts
nm: nanometers
PHA: polyhydroxyalkanoates
s.d.: standard deviation
Rf: retention factor
TAG: triacylglycerols
TLC: thin layer chromatography
WE: wax esters
w/w: weight/weight
